# Transcranial Direct Current Stimulation and Cranial Electrotherapy Stimulation in Athletes: Optimizing Athletic Performance and Sleep Quality Through Neuromodulation

**DOI:** 10.7759/cureus.116465

**Published:** 2026-09-18

**Authors:** Joanna Stezycka, Kamil Krupa, Michał Kmiecik, Franciszek Kaczmarek, Krystian Czernikiewicz, Eliza Kortus, Katarzyna Kaczmarek, Mateusz Fiutak, Sonia Marchwiak, Michalina Cuper, Paulina Cuper, Paulina Gajniak

**Affiliations:** 1 Medicine, Poznan University of Medical Sciences, Poznań, POL; 2 General Practice, Franciszek Raszeja Municipal Hospital, Poznań, POL; 3 General Practice, Poznań Voivodship Hospital, Poznań, POL; 4 Psychology, SWPS University, Poznań, POL; 5 General Practice, Poznan University of Medical Sciences, Poznań, POL; 6 General Practice, Medical University of Silesia, Zabrze, POL; 7 General Practice, Pleszew Medical Center, Pleszew, POL; 8 General Practice, Jozef Struś Municipal Multidisciplinary Hospital, Poznań, POL

**Keywords:** athletes, athletic performance, ces, neuromodulation, non-invasive neurostimulation, sleep, sleep disorders, sleep quality, sport, tdcs

## Abstract

Neuromodulation is a procedure relying on the delivery of specific stimuli to selected sites of the nervous system and hereby influencing its activity. Even though the underlying neurobiological mechanisms are not yet fully elucidated and the research is still ongoing, neuromodulation is already being used as an intervention for various mental conditions and insomnia, or it may even improve the physical performance of athletes. This narrative review summarizes current evidence on the effects of specific neuromodulation techniques on performance and sleep quality in athletes, focusing on cranial electrotherapy stimulation (CES) and transcranial direct current stimulation (tDCS). PubMed, SCOPUS, Web of Science and Ovid MEDLINE databases were searched for relevant articles. All types of publications were taken into account, but original studies, randomized controlled trials (RCTs), and meta-analyses were given priority. Current evidence shows that while tDCS may improve athletic performance, especially endurance and sport-specific skills, its impact on sleep in athletes is inconclusive. This method may increase subjectively reported total sleep time and subjective sleep quality, but no objective improvement has been demonstrated. CES findings are preliminary: evidence from the small trials suggests improvement of selected objective sleep parameters, but the evidence base is limited to two small studies, one of which lacked a sham condition. In conclusion, non-invasive neuromodulation methods, specifically tDCS and CES, may impact athletic performance and help treat sleep disorders in athletes. Neuromodulation should not be viewed as a standalone method, but rather a complementary tool to other known interventions for insomnia in athletes. Although the aforementioned techniques show promising results, the research on this topic is limited and requires further evaluation.

## Introduction and background

Neuromodulation is a targeted modification of nervous system activity through the delivery of a specific stimulus (e.g., electrical, ultrasonic, optical, magnetic, or even, in preclinical settings, genetic stimulation) to selected sites within the system [1-4]. It aims to modulate neural activity, restore impaired functions, and treat certain neuropsychiatric disorders [5,6]. Among neuromodulation techniques, non-invasive physical methods are of particular interest, including transcranial magnetic stimulation (TMS), cranial electrotherapy stimulation (CES), transcranial direct current stimulation (tDCS), and transcranial alternating current stimulation (tACS) [2-4,6]. This review focuses on publications on tDCS and CES and their influence on athletic performance and sleep quality.

Among the commonly used methods of transcranial electrical stimulation, tDCS is the most extensively investigated modality in the context of sports performance. It is a non-invasive method used for modulation of cortical excitability by applying 0.5-2 mA direct current. tDCS exerts polarity-dependent effects: anodal stimulation shifts the resting membrane potential toward the firing threshold, raising the likelihood of neuronal discharge and thus cortical excitability, whereas cathodal stimulation has the opposite, excitability-lowering effect [7,8]. During the tDCS procedure, stimulation is delivered with sponge electrodes placed on the participant’s head: the anode depolarizes neurons and increases excitability of the cortex, whereas the cathode hyperpolarizes neurons and decreases excitability [9,10]. However, the neurobiological mechanisms are not yet fully understood, despite its rapidly increasing clinical uptake and ongoing scientific research [11]. One of the non-pharmacological methods used as a helpful device for athletes at the clinical level is TMS. Specific TMS devices have been approved by the Food and Drug Administration (FDA) for use in psychiatry [12,13]. Simultaneously, the tDCS devices are starting to get acknowledged as a new solution. In 2025, the Flow FL-100 device (Flow Neuroscience AB, Malmö, Sweden), which uses tDCS, was approved by the Food and Drug Administration for home use and for the treatment of moderate to severe non-treatment-resistant depression in adult patients, both as monotherapy and as adjunctive therapy [14]. Depending on the stimulation site, tDCS has been associated with improvements in motor learning, motor performance, working memory, attention and emotional regulation, as well as changes in cerebral perfusion. However, considerable interindividual variability and methodological differences, including electrode type and montage, current intensity, stimulation duration and anatomical characteristics of participants, make it difficult to establish an optimal stimulation protocol [8].

Another neuromodulation method being investigated that may affect sleep quality and sports performance in athletes is CES. In contrast to tDCS, which relies on direct current, CES uses high-frequency pulse trains. CES is a non-invasive technique that modulates central nervous system activity and is considered a safe and accessible tool for treating mental disorders [15]. CES devices deliver low-intensity pulsed currents, typically in the microampere range rarely exceeding 4 mA, thus posing a low risk of adverse effects [16-18]. During the procedure, electrodes are typically clipped to the earlobes or placed over the mastoid processes, the temples, or the supraorbital region [15,19]. The current transmitted through the electrodes penetrates soft tissue and bone to certain extent, reaching the cerebral cortex and subcortical structures, affecting local blood flow and the electrical activity of the brain [19,20]. Despite the long record of CES utility in psychiatry, its mechanism of action has not been fully elucidated. Various explanations of the influence on the central nervous system have been proposed, including alteration of neurotransmitter release, modification of cell membrane potential, cell excitability, and neuromodulation of subcortical structures [21-23]. The electroencephalography studies indicate the effect of CES on the electrical activity of the brain, including an increase in alpha-band power, which is associated with cognitive functions and memory [21,24]. Meta-analytic data suggest that CES may reduce depressive and anxiety symptoms [25]; however, a critical review has emphasized substantial methodological limitations of the underlying trials, so this connection is not robust as lacking conclusive evidence [21]. Furthermore, several studies have shown that CES may reduce sleep latency, increase sleep duration, improve sleep quality, and reduce subjective daytime drowsiness [15,18,26]. Although the efficacy of CES for insomnia itself remains unconfirmed, it improved mood and subjective well-being in individuals with subclinical insomnia [27]. Insomnia and poor sleep are common in athletes; pre-competition stress and anxiety impair sleep, and insufficient sleep may in turn compromise mood and performance [28].

## Review

Materials and methods

This narrative review compiles the current knowledge on the role of neuromodulation in sports performance and sleep disorders. The narrative form was chosen in order to integrate data related to the mechanisms of action of neuromodulation with clinical data. The neuromodulation modalities considered were tDCS and CES. Four researchers were delegated to conduct the research of databases. Each database was searched by all four researchers, who strictly followed the inclusion and exclusion criteria. The databases were searched in June 2026 by title and abstract using search strings in which the keywords were combined with the Boolean operators AND and OR: "cranial electrotherapy stimulation", "transcranial neurostimulation", "transcranial direct current stimulation", "sleep", "insomnia", "athletes", "sports". No date restrictions were applied, in order to maximize the amount of literature included. All types of publications were taken into account, but original studies, including randomized controlled trials (RCTs), systematic reviews and meta-analyses were prioritized. The inclusion criteria were adult humans and studies of athletes assessing physical performance or sleep parameters and studies of tDCS/CES for insomnia or sleep disturbance in general adult populations, which were used solely for the sleep section. The exclusion criteria were paediatric populations and animal studies. As a result of this targeted search, 781 records were obtained from PubMed (n = 145), SCOPUS (n = 180), Web of Science (n = 256) and Ovid MEDLINE (n = 200). Title and abstract screening yielded all articles of which 72 articles qualified for full-text assessment. Twenty-nine articles met inclusion criteria during full-text assessment. Publications that were citation-tracked or were relevant in terms of a significant contribution of knowledge to the given subject, key findings, and mechanisms allowing an understanding of the issues addressed in this narrative review were also added manually (n = 10). Articles identified as duplicates from different database searches were removed. Ultimately, 39 publications were selected and synthesized, taking into account the most important mechanisms and the clinical and epidemiological data. The search strategy, including databases, search algorithm, filters and limits, and retrieved record number, is presented in Table 1. The search strategy is depicted also as a flow diagram in Figure 1. The inclusion and exclusion criteria used during the literature selection process are displayed in Table 2.

**Table 1:** Search strategy.

Database	Search Algorithm	Filters and Limits	Retrieved Records
PubMed	(“cranial electrotherapy stimulation” OR “transcranial neurostimulation”) AND (sleep OR insomnia) AND (athletes OR athlete OR sport OR sportsmen OR sports OR athletics OR player OR players OR “elite athletes” OR “competitive athlete” OR sportswoman OR sportsman) (“cranial electrotherapy stimulation” OR “transcranial neurostimulation”) AND (athletes OR athlete OR sport OR sportsmen OR sports OR athletics OR player OR players OR “elite athletes” OR “competitive athlete” OR sportswoman OR sportsman)	Title/Abstract; no date restrictions; free full-text; English language	n = 145
SCOPUS			n = 180
Web of Science			n = 256
Ovid MEDLINE			n = 200

**Figure 1:**
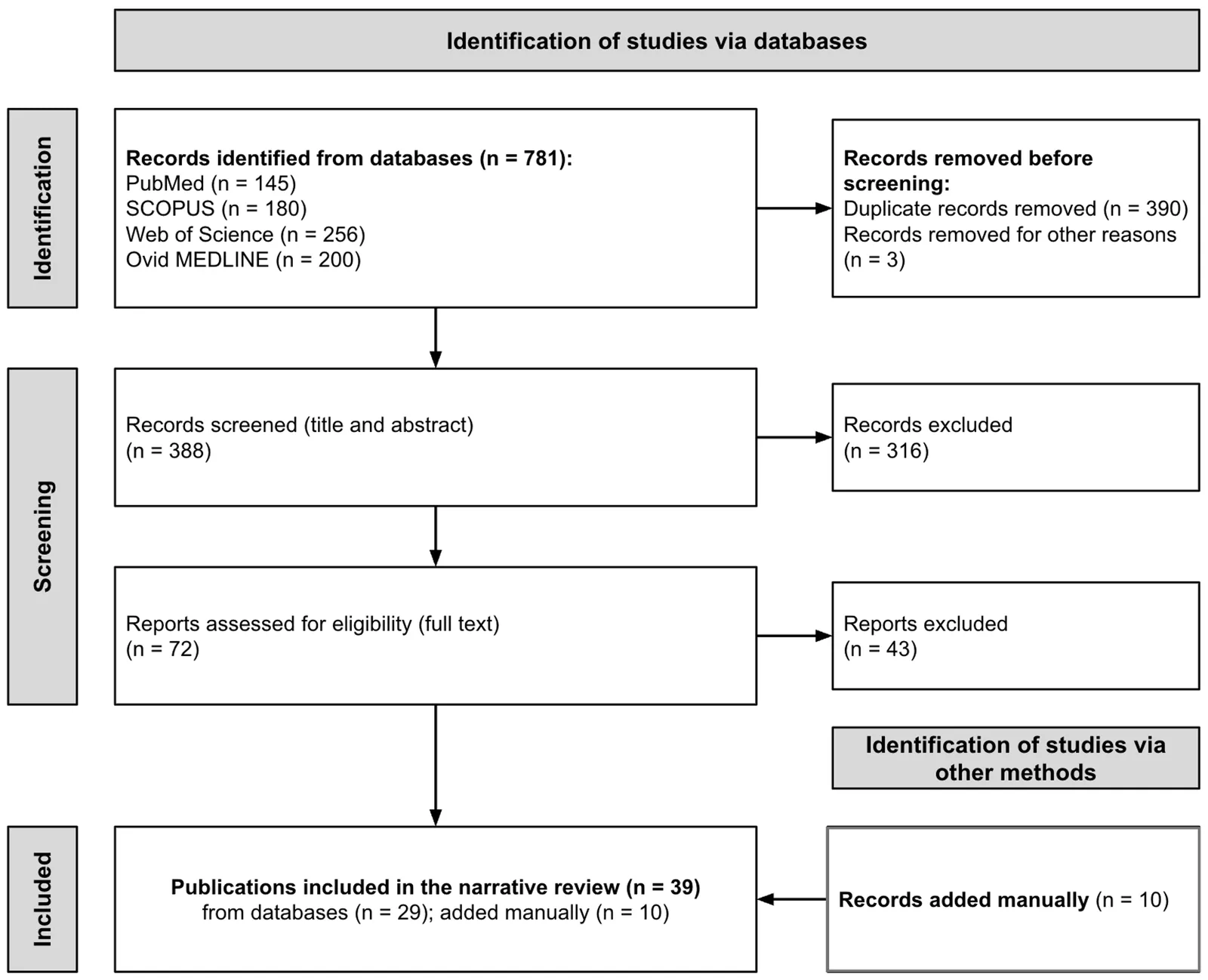
Flow diagram of the literature search and selection process.

**Table 2:** Inclusion and exclusion criteria.

Inclusion	Exclusion
Adult humans (≥ 18 years)	Paediatric populations
Transcranial direct current stimulation (tDCS), Cranial electrotherapy stimulation (CES)	Other neuromodulation techniques
Studies in athletes evaluating physical performance and sleep quality	Animal studies
Studies in general population on insomnia or sleep disturbance	

Owing to the narrative-review format, this work is burdened with unavoidable limitations. Publications were selected on the basis of relevance rather than a formal systematic-review protocol. In order to mitigate, though not eliminate, this risk, evidence from randomized controlled trials and meta-analyses was prioritized, and null findings were reported alongside positive ones. A notable limitation of the reviewed literature is the small sample size of the individual trials (n = 12-40), which diminishes effect size precision and increases predisposition to selection bias. Given these small cohorts, the included studies present an elevated risk of bias, which was accounted for during the qualitative synthesis. Also, cautious interpretation of the studies was repeatedly emphasized, especially when the results presented borderline statistical significance.

tDCS in athletic performance

The Specificity Model: Primary Motor Cortex (M1) vs Dorsolateral Prefrontal Cortex (DLPFC)

A comprehensive understanding of the mechanisms underlying tDCS, its potential benefits, and its optimal application requires consideration of the methodological specificity of this form of stimulation. The effects of tDCS appear to depend substantially on the selected stimulation site, making the mapping of stimulation targets to specific performance outcomes a central issue [29,30]. Protocols targeting the dorsolateral prefrontal cortex (DLPFC) were more strongly associated with perceived recovery, cognitive performance, and sport-specific skill execution, whereas stimulation of the primary motor cortex (M1) was more frequently linked to motor adaptations [29]. In addition, electrode montage and the current return pathway also appear to be important determinants of the stimulation outcome. The most pronounced benefits were observed when electrode placement and stimulation timing were aligned with the presumed neural substrates underlying the target function. In contrast, nonspecific or poorly matched protocols often yielded null effects and, in some cases, undesirable behavioural consequences. Together with an appropriate stimulation protocol, these factors may constitute prerequisites for achieving the maximal benefit from tDCS [29]. A potential explanation for this principle, as well as for the observation that athletes derive greater benefits from stimulation than non-athletes, is hypothesized to be the notion that tDCS strengthens existing, well-established neural networks rather than creating new neuronal connections [30,31]. In some soccer-specific studies, tDCS protocols administered 12-48 hours after a match were associated with lower perceived fatigue and improvements in selected subjective recovery outcomes, including reduced muscle soreness, better perceived recovery, and improved sleep quality. Although a single tDCS session generally produced inconsistent effects on strength and endurance [29,31], individual studies have reported acute improvements in explosive performance following M1 stimulation [30]. By contrast, repeated M1 stimulation administered concurrently with training appeared to exert beneficial effects on functional performance, as reflected by improvements in vertical jump height and sprint performance [29]. Furthermore, combining tDCS with high-intensity interval training (HIIT) was associated with enhanced aerobic capacity, including increases in maximal oxygen uptake (VO₂max) [29].

Pooled Evidence on Physical Performance and Application of tDCS in Specific Sport Disciplines

Based on recent meta-analyses, the current evidence supporting the benefits of tDCS should be considered moderate, and the effect of this stimulation method appears to be statistically significant. Jiang et al. reported a statistically significant, moderate pooled effect of acute anodal tDCS on sport-specific performance (SMD = 0.39, 95% CI: 0.23-0.54), with significant subgroup effects for endurance, primarily reflected by longer time to exhaustion, and competitive-collaborative skill tasks but not for strength or precision-skill outcomes [32]. The stimulation applied in the analysed studies was exclusively tDCS and, in most cases, targeted mainly the primary motor cortex (M1), prefrontal cortex (PFC), temporal cortex (TC) and cerebellum (CB); statistically significant pooled effects were observed only for M1 and PFC stimulation, although differences between the stimulation-site subgroups were not significant [32].

A broader meta-analysis conducted by Liu et al. included 40 studies with a total of 778 participants (both athletes and non-athletes) [31]. The analysis demonstrated a statistically significant, although small overall effect of tDCS in both groups. The greatest benefits were shown in terms of improvements in muscular and cardiopulmonary endurance, as well as static balance. The magnitude of improvement was, however, greater in athletes than in non-athletes. The authors themselves note that this result is inconsistent with their initial assumption of a ceiling effect in athletes, given that individuals with superior motor skills are thought to possess more efficient cortical functioning and require lower levels of cortical activation [31]. In contrast, other authors have proposed that tDCS may exert greater effects in athletes because it primarily strengthens pre-existing neural networks, presumed to be more highly developed in trained individuals, through long-term synaptic potentiation [30,33]. However, these mechanisms have not been directly demonstrated, and apparent differences between athletes and non-athletes may also reflect variation in tasks, stimulation protocols, baseline performance, and study design [30,31,33,34].

The current evidence suggests that the effects of tDCS in athletes may be sport-specific and largely depend on the cognitive and physiological demands characteristic of a given discipline. Although the reported effects are generally modest and heterogeneous, the available studies suggest that tDCS may enhance selected aspects of performance when appropriately matched to the target function and training context [8,34]. In sports requiring a high degree of cognitive control and psychological resilience, such as collegiate volleyball, repeated stimulation of DLPFC was associated with reduced pre-competition anxiety and improvements in selected cognitive functions [35]. In another review synthesizing evidence in basketball players stimulated with tDCS in M1 region, psychological fatigue and cognitive outcomes remained limited and inconsistent; however, M1 stimulation was associated with improvements in motor performance, such as improved shooting accuracy, shorter dribbling time or repeated-sprint ability [34]. In combat sports, where rapid information processing and visuomotor responses are essential, more pronounced effects have been reported. In experienced taekwondo athletes, simultaneous stimulation of M1 and the thoracolumbar (T12-L2) spinal segments improved selective attention and reaction time, without improvement of visuospatial working memory and sport-specific performance outcomes (endurance time, number of kicks) [33]. Comparable findings were obtained in experienced boxers, in whom repeated sessions of concurrent M1 and spinal direct current stimulation resulted in faster hand reaction times and improved selective attention, highlighting the possible role of corticospinal mechanisms in sports relying on rapid upper-limb responses [36]. Both randomized trials had very small samples (15 taekwondo practitioners and 14 boxers, respectively) and used dual-mode stimulation, thus observed effects cannot be attributed specifically to M1 stimulation. In soccer, acute performance enhancement appears to be limited. A single session of bihemispheric M1 stimulation failed to improve countermovement jump performance, heart rate recovery, perceived exertion, muscle soreness, or subjective recovery indices in amateur players, suggesting that isolated stimulation may not be sufficient to produce measurable benefits in this population [37]. However, broader evidence from soccer-specific reviews suggests that repeated and task-oriented protocols may facilitate cognitive performance, recovery perception, and sport-specific skill execution, particularly when stimulation is combined with training [29]. Table 3 provides an illustrative overview of studies that examined tDCS within individual sport disciplines; works addressing multiple sports at once, including meta-analyses, were not included, as the table is not intended as a comprehensive review of all available data.

**Table 3:** Sport-specific applications and effects of transcranial direct current stimulation (tDCS) in athletes: an illustrative overview of single-discipline studies. tDCS: transcranial direct current stimulation; tsDCS: transcutaneous spinal direct current stimulation; DLPFC: dorsolateral prefrontal cortex; M1: primary motor cortex; T12-L2: 12th thoracic to second lumbar vertebrae (thoracolumbar region); RCT: randomized controlled trial; BAI: Beck Anxiety Inventory; DSST: Digit Symbol Substitution Test; EMG: electromyographic (biofeedback); RT: reaction time; CMJ: countermovement jump; HR: heart rate; RPE: rating of perceived exertion; VAS: Visual Analogue Scale; SRS: Subjective Recovery Scale; CK: creatine kinase; ns: non-significant; CI: confidence interval; d: Cohen’s d. *Data was obtained from small subgroups and should be interpreted with caution.

Author (year) [ref.]	Sport	Study design and sample	Stimulation (target and method)	Stimulation parameters	Main results	Effect size and 95% CI	p-value
Joshi et al. (2025) [35]	Volleyball (collegiate athletes)	RCT, parallel-group, sham-controlled (participant-blinded); n = 19 (randomized); n = 12 (analysed 6 vs 6)	Anodal tDCS over the left DLPFC; cathode over the right supraorbital region	2 mA; 20 min/session for 10 consecutive days (multi-session)	Improved: pre-competition anxiety (BAI), Stroop, DSST, EMG relaxation	Effect size (d)*: Stroop: 1.79; DSST: 0.56; EMG: 0.46	BAI: 0.04; Stroop: 0.01; DSST: 0.01; EMG: 0.02
						95% CI: Stroop: 1.31-8.02; DSST: 2.67-21.32; EMG: 71.6-747.73; BAI: IQR: 15.25-23.75)	
Kamali et al. (2023) [33]	Taekwondo (elite athletes)	Single-arm, double-blind, real-vs-sham crossover; n = 15 (14 analysed)	Dual-mode: anodal tDCS over M1 + cathodal tsDCS over the thoracolumbar region (T12-L2)	2 mA; 13 min single real session (crossover, 72 h apart)	Improved: selective attention (+31%), reaction time (+4.7%)	Effect size (d): selective attention 1.84; reaction time: 0.02	Selective attention < 0.0001; reaction time < 0.0001; others > 0.05 (ns)
					No change: visuospatial memory, endurance time, number of kicks	95% CI: not reported	
Kamali et al. (2021) [36]	Boxing (experienced athletes)	Real-vs-sham crossover; n = 14	Dual-mode: anodal tDCS over M1 hand area (C3/C4) + cathodal paraspinal tsDCS (hand-corresponding region)	2 mA; 13 min single real session (crossover, 72 h apart)	Improved: right- and left-hand reaction time, selective attention (-47.5% errors)	Effect size, 95% CI: not reported	Right-hand RT < 0.0001; left-hand RT < 0.0006; selective attention < 0.0003
					No change: cognitive/visuospatial memory		
Rocha et al. (2024) [37]	Soccer (amateur players)	Cross-sectional, randomized, triple-blind, controlled; n = 27 (3 groups × 9: active/sham/control)	Bi-hemispheric anodal tDCS over M1 (cephalic montage)	2 mA; 15 min single session	No change: CMJ, heart-rate recovery, RPE, muscle soreness (VAS), subjective recovery (SRS)	Not applicable	All > 0.05

Conclusion and Practical Implications

The effects of tDCS are highly dependent on the interaction between stimulation parameters, the characteristics of the athlete, and the specific motor or cognitive demands of the task. Whether the reported changes translate into meaningful competitive advantages remains to be demonstrated. Because active tDCS produces perceptible cutaneous sensations, blinding integrity is a recurrent concern in sham-controlled trials, and positive findings in small sports-science samples may be inflated by publication bias; both issues warrant caution when interpreting the evidence summarized here. Therefore, tDCS should be considered a complementary and highly individualized tool rather than a universal method for enhancing sports performance [8,29,31].

The role of CES and tDCS in sleep quality in athletes

Sports medicine specialists recognize the necessity of non-pharmacological methods that enhance sleep quality and treat sleep disturbances in athletes. One such method is neuromodulation. Some studies suggest that the use of different neural stimulation techniques may enhance sleep quality and sleep efficiency [22,38,39], albeit randomized tDCS data do not indicate objective improvements [9,10]. The neuromodulation methods could be most beneficial in the period before competition, when stress, anxiety and other negative emotions accumulate [28]. The comparison of sleep-specific applications and effects of CES and tDCS in athletes is displayed in Table 4.

**Table 4:** Sleep-specific applications and effects of cranial electrotherapy stimulation (CES) and transcranial direct current stimulation (tDCS) in athletes: a comparison of studies.

Selected neuromodulation technique	Author (year) [ref.]	Intervention	Key outcomes	Sample size	Major limitations
CES	Chang et al. (2022) [22]	2-week CES intervention vs sham control	Reduction of negative mood states (anger, tension, depression), shortened choice reaction time, attenuated pre-competition decline in sleep efficacy	n = 40 collegiate athletes with poor sleep quality (n = 20 active CES; n = 20 sham control)	Small sample size, brief 2-week intervention duration, lack of long-time follow up
	Tan et al. (2023) [38]	5-day CES intervention vs sham control	improved subjective sleep quality (in both CES group and sham control group), reduced actigraphy-measured wake after sleep onset, increased objective sleep efficiency	n = 25 (enrolled); n = 18 (analysed) (n = 9 active CES; n = 9 sham control)	Small sample size, brief 5-day intervention duration, lack of long-time follow-up
	Tsou et al. (2025) [39]	4-week CES intervention vs CBT-I	improved subjective sleep quality and daytime sleepiness (in both CES group and CBT-I group), deeper sleep (reduced light-sleep and increased deep-sleep percentages) with changes in nocturnal heart-rate variability	n = 24 athletes with poor sleep quality (n = 12 active CES; n = 12 CBT-I)	Small sample size, absence of sham/control group, lack of long-time follow up
tDCS	Charest et al. (2021) [9]	5-night tDCS intervention (2 sessions) vs sham control	Improved subjective sleep quality, improved subjective total sleep time, no significant objective improvement after active tDCS	n = 30 collegiate student-athletes (n = 15 active cathodal tDCS; n = 15 sham control)	Small sample size, brief intervention duration, reliance on an in-laboratory setup
	Charest et al. (2020) [10]		No significant objective effect on sleep depth as measured by the odds ratio product (ORP)		

Cranial Electrotherapy Stimulation (CES)

CES is used in mental disorders [15], and studies suggest it may alleviate anxiety and depressive symptoms [25]. CES use may have an impact on cognitive functions and memory [21,24]. Moreover, CES appears to have a range of positive effects on overall sleep in athletes. Although different approaches were taken, all of the studies discussed below suggest that CES may be beneficial for sleep in athletes [22,38,39]. Tan et al. conducted a pilot study of 25 professional athletes with poor sleep quality (12 receiving CES for 30 minutes on five consecutive days; 13 controls receiving no intervention) [38]. The study showed that CES reduced actigraphy-measured wake after sleep onset and increased objective sleep efficiency, whereas total sleep time did not change significantly and subjective sleep quality improved in both groups. The absence of a sham condition means expectation effects cannot be excluded [38]. In another study, Tsou et al. directly compared four weeks of CES (Alpha-Stim (Electromedical Products International Inc., Mineral Wells, USA), 100 µA, 60 min, three times per week) with Cognitive Behavioral Therapy for Insomnia (CBT-I) in 24 combat-sport athletes with poor pre-competition sleep quality [39]. Subjective sleep quality, measured by the Pittsburgh Sleep Quality Index (PSQI), and daytime sleepiness, measured by the Epworth Sleepiness Scale (ESS), improved in both groups [39]. Sleep efficiency increased significantly only after CBT-I, whereas CES was associated with a shift towards deeper sleep (reduced light-sleep and increased deep-sleep percentages) and with changes in nocturnal heart rate variability. The study lacked a no-treatment control group, so placebo effects cannot be excluded. These findings support the view that CES may complement, rather than replace, CBT-I in athletes [39]. In a randomized, sham-controlled study of 40 sport-university athletes tested during the pre-competition period, two weeks of CES significantly reduced negative mood states (anger, tension, depression) and shortened choice reaction time. Actigraphy-measured sleep efficiency declined as the competition approached; the decline was attenuated in the CES group, but sleep efficiency did not improve above baseline [22]. The authors of these studies emphasized the need for further research of CES interventions on sleep quality in athletes.

Transcranial Direct Current Stimulation (tDCS)

Apart from its use as a non-pharmacological treatment in psychiatry [14], tDCS may improve motor learning, motor performance, working memory, attention and emotional regulation in athletes [8]. In addition, tDCS may have an impact on subjective total sleep time. Charest et al. conducted a randomized, sham-controlled study of the effect of tDCS on the sleep quality of student-athletes [9]. They recruited 30 participants (15 males, 15 females) with PSQI > 5 and Insomnia Severity Index (ISI) > 7, and randomized them into experimental and control groups (15 athletes each). The experimental protocol consisted of five nights. One adaptation night was followed by two nights of baseline polysomnography. During two final nights, bifrontal tDCS (2mA, 20 min, polarity and electrode montage as specified in the source) targeting the "top-down" pathway of sleep regulation was delivered 90 minutes before bedtime. The control group received sham stimulation. Psychomotor Vigilance Test (PVT) was administered pre- and post-intervention to assess the impact of tDCS on cognitive performance. Mean reaction time in PVT decreased after the stimulation, but there was no significant difference between experimental and control groups. Sleep-diary data indicated a marginal increase in subjective total sleep time, whereas polysomnographic analyses showed no significant objective improvement after active tDCS. In the tDCS group, PSQI, ISI and Athlete Sleep Screening Questionnaire (ASSQ) results showed statistically significant improvement post-intervention when compared with the baseline values. However, only PSQI and ASSQ post-treatment scores were significantly better than in the sham stimulation group [9]. Odds ratio product (ORP)-based sleep-depth outcomes, calculated from polysomnographic data, failed to show any significant benefit of tDCS. Interestingly, men in the experimental group increased their time spent at ORP values of 0-1 by 41.57 minutes, while women decreased by 11.03 minutes on average. Time spent in lower ORP index (0-1) is related to better sleep quality [10]. In conclusion, tDCS may increase total sleep time and improve subjective sleep quality, but no objective effect was observed. Further research is required to verify if tDCS is beneficial for athletes with sleep disorders, but current evidence is not supportive enough to recommend it in this context.

## Conclusions

Non-invasive neuromodulation techniques, in particular tDCS and CES, seem to be a promising option for sports medicine specialists. Both techniques are generally well tolerated; the most common adverse events of tDCS are transient tingling, itching, skin redness and headache, with rare skin burns under the electrodes, and low-intensity CES has a similarly benign profile. Nevertheless, safety data in athletic populations are scarce and should be collected systematically. These techniques may influence both athletes’ performance and crucial recovery processes like sleep architecture. Neuromodulation effects are target-specific and can differ in various sport disciplines. The effect of tDCS depends on the targeted stimulation site. Different electrode localizations have been associated with, among others, better motor learning and performance, attention, and emotional regulation. Preliminary evidence suggests that tDCS may be used as a supportive tool for reducing pre-competition anxiety. Its protocols also target different areas of athletes’ performance. Protocols targeting M1 appear to be most consistently associated with improvements in motor function, such as improved shooting accuracy, dribbling time, or repeated sprints. In contrast, stimulation of DLPFC was linked to potential reduction of anxiety and better selected cognitive functions. CES and tDCS may have a positive effect on athletes with sleep disorders. CES may positively affect some objective sleep parameters and may reduce awake time before falling asleep, but the effects observed to date are moderate. This method may also reduce sleep deterioration observed during the pre-competition period in athletes. The effects of tDCS on sleep in athletes remain understudied; to date, a single randomized trial has shown improvement of total sleep time and subjective sleep quality (measured by PSQI, ISI and ASSQ questionnaires), but no objective benefits were observed in conventional polysomnographic data or ORP. Neuromodulation techniques should not be treated as an autonomous solution for athletes’ problems. Rather, they should be an addition to other known methods like CBT-I for managing insomnia in athletes. Although the neuromodulation techniques appear to be promising approaches in athletic performance enhancement and sport-related sleep disturbances, further research in this field is necessary. Our knowledge of neuromodulation in athletes is limited by small sample sizes or heterogeneity of the athlete populations. Further research is needed to establish neuromodulation as a safe and ethical tool. The integration of aforementioned neuromodulation techniques into athletic training raises questions about the competitive fairness and equal opportunity. The issues of unequal access to the necessary equipment, patented stimulation protocols and specialized experts should be discussed thoroughly. Another concern lies in the lack of detectable physiological markers or biological trace in blood or urine of athletes which makes it impossible to detect. To this end, reliable double-blind randomized controlled trials with standardized parameters of neuromodulation techniques are needed.
